# Editorial: Clinical cancer research in vulnerable populations

**DOI:** 10.3389/fonc.2023.1166714

**Published:** 2023-03-02

**Authors:** Laura Tack, Patricia Schofield, Tom Boterberg, Christopher N. Parris, Philip R. Debruyne

**Affiliations:** ^1^ Kortrijk Cancer Centre, Department of Medical Oncology, General Hospital Groeninge, Kortrijk, Belgium; ^2^ Department of Human Structure and Repair, Ghent University, Ghent, Belgium; ^3^ School of Nursing and Midwifery, Faculty of Health, University of Plymouth, Plymouth, United Kingdom; ^4^ Medical Technology Research Centre (MTRC), School of Life Sciences, Faculty of Science and Engineering, Anglia Ruskin University, Cambridge, United Kingdom

**Keywords:** vulnerable patient populations, cancer, cancer research, clinical trials, eligibility criteria, minority populations

Vulnerable populations in cancer care include a wide array of possible conditions (see **Table 1**). Their vulnerabilities, whether medical, sociocultural, age- or socioeconomic-related, cause these cancer populations to be excluded from clinical trials. This introduces bias and a Matheus effect as clinical trial results are often not fully representative of the whole target population. This underrepresentation of the majority of cancer patients in clinical trials is a major drawback.

**Table 1:** Vulnerable populations.

Addicts; Drug or substance abusers (1)
Adolescents and young adults (AYA)
Brain and leptomeningeal metastases (2)
Children; minors https://www.frontiersin.org/articles/10.3389/fonc.2022.882501/full
Dementia sufferers; Cognitively impaired (3)
Detainees; Prisoners
Geographic (rural) isolation https://www.frontiersin.org/articles/10.3389/fonc.2022.871192/full
LGBTQ+ patients
Migrant populations; Ethnic minorities; Indigenous people (4) https://www.frontiersin.org/articles/10.3389/fonc.2022.997158/full
Older patients; geriatrics (5)
Organ dysfunction sufferers (chronic renal impairment, hepatic impairment, kidney failure, hemodialysis, liver failure)
Patients who received too many (or too few) lines of treatment https://www.frontiersin.org/articles/10.3389/fonc.2022.1027353/full
Patients with auto-immune diseases https://www.frontiersin.org/articles/10.3389/fonc.2022.922579/full
Patients with chronic viral infections (HIV, HBV, viral hepatitis, etc.) https://www.frontiersin.org/articles/10.3389/fonc.2022.957325/full
Patients with poor performance status
Patients with prior or concurrent malignancies
People with limited health literacy
Physically handicapped patients; Obese patients; Cachexia sufferers
Pregnant patients
Psychiatric comorbidities; Psychiatric patients
Socioeconomically deprived; Socially disadvantaged populations; Social isolation; Remote isolation
Solid organ transplant recipients
Veterans

The treatment of cancer patients with surgery, radiotherapy, and systemic anticancer drugs has reached an increasingly high level of effectiveness, sometimes shifting a cancer diagnosis towards the possibility of living a long life with a chronic cancer therapy treatment that takes place on a regular basis over a long period of time. Moreover, research is often so advanced that we may discuss “personalised cancer medicine” for different cancer types.

Unfortunately, the progress that cancer research has made in cancer prevention, early detection, and treatment is not equitably accessible and applicable to vulnerable cancer patient populations. The main reason is that the patients included in clinical trials are not always representative of the whole target population. Indeed, the generalizability of trial results to all patients is usually hampered by the strict inclusion criteria of the clinical trials, leading potentially to overinflated reported benefits. In addition, the toxicity may be substantially higher in these vulnerable populations. Therefore, patients from the groups listed in **Table 1** may not receive the best treatment option for their condition. Furthermore, the ethical implications of including vulnerable populations in clinical trials are often insurmountable for researchers attempting to gain approval for these studies.

A recent report by the ASCO focused on the necessity of an equal opportunity to participate in clinical trials and research in general. They proposed many strategies to improve equity in cancer research, such as broadening eligibility criteria (6). The need to revise these strict clinical trial eligibility criteria was seconded by Riner et al., pointing out to differentially excluding Black patients from participating in pancreatic ductal adenocarcinoma clinical trial candidacy. Restrictive criteria enhance diseases that are more prevalent in minority populations, such as Black and Hispanic, to not be investigated in the populations affected (4). Recently, the FDA made a report advocating the broadening of cancer trial eligibility criteria. They provided recommendations regarding the inclusion of patients with brain metastases, urging a safe and effective use of products that concerns the patient population that will be prescribed the drug in clinical practice (2).

Next to race and ethnicity, age has also been identified as a strict eligibility criterion leading to the underrepresentation of older patients with cancer in clinical trials. Older patients are not only numerically underrepresented in clinical trials, but the large heterogeneity within this population also causes the older patients included in clinical trials to be fitter than their real-life counterparts. The lack of evidence-based data related to the benefits and risks of cancer treatment in vulnerable older patients limits the applicability of trial results within the field of oncology to older patients with cancer who may have a vulnerable or frail profile in particular. Indeed, not automatically excluding patients of older age, or with lower ECOG Performance Status, would make trial results more reliable for the vulnerable older cancer patient population who actually represents the majority of cancer diagnoses and deaths. We have to acknowledge that there are also good reasons to exclude vulnerable patients from cancer clinical trials as cancer treatments may cause unique toxicities because of underlying conditions such as comorbidities. Other physiologic changes, such as reduced renal function, may also impact chemotherapy dosing for example. However, to deliver high-quality cancer care to vulnerable cancer patient populations, more research targeting this evidence gap will be required. In a previous study by our research group, we made suggestions for new trial designs to allow more vulnerable patients to receive upfront dose reductions or less intense regimens and stressed the concept of a dose-expansion cohort (DEC) dedicated to vulnerable older patients to be incorporated in phase 1b/2a protocols (5).

The extrapolation of clinical trial results to a real-life population involves the risk of exposing many patients to strategies that have never proven to be effective in underrepresented subpopulations. The recently developed combination therapies involving immune checkpoint inhibitors have potentially serious immune-related and other adverse events, which might have a greater impact on older and more vulnerable patients. An upcoming phase IV clinical trial by our research group entails the investigation of the safety and tolerability of the first-line combinations of a PD-1 inhibitor with a CTLA-4 or tyrosine kinase inhibitor (TKI) in older patients with stage IV kidney cancer (mccRCC). We will include four currently approved first-line immunotherapy combination regimens in eight cohorts of fit and vulnerable older patients with mccRCC and compare the safety profiles of the cohorts to the reported proportions of the respective landmark trials (7). We expect that the observed toxicity in cohorts of fit older patients will be in line with the published data. We hypothesize that we may observe clinically relevant excessive toxicity in vulnerable older patients with mccRCC.

The outcomes of such post-marketing observational studies, whether reported adverse events leading to treatment discontinuation in older patients are maintained in real-life practice, are also of interest for the financial aspect of (phase IV) clinical trials. Cost-utility analysis results and health technology assessments (HTH) may not apply to vulnerable populations. Moreover, inadequate treatments pose a logistic and financial risk to the health systems.

In conclusion, we want this special issue to serve as a tribute to cancer research groups who have made efforts to focus their research on vulnerable cancer patient populations. We hope the issue can serve as a motivation for others to pursue this type of research or pursue their current intentions and address the needs of vulnerable patient populations. We advocate for more inclusive criteria, giving access to vulnerable patient populations and thereby reducing disparities in clinical trial participation.

## Author contributions

LT, PS, TB, CP, and PD designed and wrote this editorial article. All authors contributed to the article and approved the submitted version.
